# HPLC-MS Profiling, Antioxidant, Antimicrobial, Antidiabetic, and Cytotoxicity Activities of *Arthrocnemum indicum* (Willd.) Moq. Extracts

**DOI:** 10.3390/plants11020232

**Published:** 2022-01-17

**Authors:** Hafedh Hajlaoui, Soumaya Arraouadi, Hedi Mighri, Siwar Ghannay, Kaïss Aouadi, Mohd Adnan, Abdelbaset Mohamed Elasbali, Emira Noumi, Mejdi Snoussi, Adel Kadri

**Affiliations:** 1 Research Unit Valorization and Optimization of Resource Exploitation (UR16ES04), Faculty of Science and Technology of Sidi Bouzid, University of Kairouan, Campus University Agricultural City, Sidi Bouzid 9100, Tunisia; 2 Regional Center of Agricultural Research (CRRA) Sidi Bouzid, Gafsa Road Km 6, PB 357, Sidi Bouzid 9100, Tunisia; bio.soumaya@gmail.com; 3 Laboratory of Valorization of Unconventional Waters, INRGREF, University of Carthage, Road Hedi EL Karray, El Menzah IV, PB 10, Ariana 2080, Tunisia; 4 Range Ecology Laboratory, Arid Region Institute, University of Gabes, El-Jorf Road Km 22.5, Medenine 4119, Tunisia; mighrih@yahoo.fr; 5 Department of Chemistry, College of Science, Qassim University, Buraidah 51452, Saudi Arabia; S.ghannay@qu.edu.sa (S.G.); k.aouadi@qu.edu.sa (K.A.); 6 Faculty of Sciences of Monastir, Avenue of the Environment, University of Monastir, Monastir 5019, Tunisia; 7 Department of Biology, College of Science, University of Hail, P.O. Box 2440, Ha’il 81451, Saudi Arabia; drmohdadnan@gmail.com (M.A.); emira_noumi@yahoo.fr (E.N.); snmejdi@yahoo.fr (M.S.); 8 Clinical Laboratory Science, College of Applied Medical Sciences-Qurayyat, Jouf University, Sakaka P.O. Box 2014, Saudi Arabia; 9 Laboratory of Bioressources: Integrative Biology and Recovery, High Institute of Biotechnology-University of Monastir, Monastir 5000, Tunisia; 10 Laboratory of Genetic, Biodiversity and Valorization of Bioressources, Higher Institute of Bio-Technology of Monastir, University of Monastir, Avenue Taher Hadded BP 74, Monastir 5000, Tunisia; 11 Faculty of Science and Arts in Baljurashi, Albaha University, P.O. Box 1988, Albaha 65527, Saudi Arabia; lukadel@yahoo.fr; 12 Department of Chemistry, Faculty of Science of Sfax, University of Sfax, B.P. 1171, Sfax 3000, Tunisia

**Keywords:** *Arthrocnemum indicum* extracts, halophytes, phytochemicals, antimicrobial, antioxidant, antidiabetic, cytotoxicity

## Abstract

The purpose of this study was to evaluate for the first time the phytochemical constituents and biological properties of three (ethanol, acetone, and hexane) *Arthrocnemum indicum* (Willd.) Moq. (*A. indicum*) extracts. Quantitative analysis revealed the significantly (*p* < 0.05) dominance of ethanolic extract on total polyphenol (TPC; 303.67 ± 4.16 mg GAE/g DR) and flavonoid (TFC; 55.33 ± 2.52 mg CE/g DR) contents than the other extracts, also displaying high and equipotent condensed tannin (TCTC) contents as the acetone extract. The qualitative HPLC-MS analysis elucidates 19 and 18 compounds in ethanolic and acetonic extracts, respectively, belonging to the phenolics and flavonoids chemical classes. The extracts were also screened for their in vitro antioxidant activities using 1,1-diphenyl-2-picrylhydrazyl, superoxide anion, and ferric ion (Fe^3+^) reducing antioxidant power (FRAP), demonstrating the potent antioxidant activity of ethanolic extract, due to its stronger scavenging DPPH^•^ (IC_50_ = 7.17 ± 1.26 μg/mL) which is not significantly (*p* > 0.05) different from the positive control, BHT (IC_50_ = 10.70 ± 0.61 μg/mL), however moderate activity through FRAP and superoxide anion radicals have been observed. Four Gram-positive, four Gram-negative bacteria, and four pathogenic fungi were used for the antimicrobial activity. In addition, *S. epidermidis, M. luteus, E. faecalis, C. glabrata, C. parapsilosis, C. krusei* were found to be the most susceptible strains towards ethanolic extract. Cytotoxicity values against human colon adenocarcinoma cells (HT29) and human epidermoid cancer cells (Hep2), and one continuous cell lineage control (Vero) revealed that the HT29 cancer cell line was the most responsive to *A. indicum* shoot extract treatment and significantly (*p* < 0.05) different from the other cancer cells. Moreover, when tested for their antidiabetic inhibitory effect, ethanol extract recorded the highest antidiabetic effect with IC_50_ = 13.17 ± 1.04 mg/mL, which is 8.4-fold higher than acetone extract. Therefore, the present study provides new findings on the use of *A. indicum* shoot ethanolic extract to cure many incurable diseases.

## 1. Introduction

Aromatic medicinal plants (AMP) with multiple targets might play a role in drug discovery and development due to their potential health-promoting effects and are a source of new pharmaceutical substances [1,2,3]. Herbal extracts and their phytochemicals have been extensively used in folkloric medicine to cure, heal, or reduce the aggressiveness of disease and treat various ailments and health disorders [4,5,6,7]. Among them, halophyte plants, known for their high salt tolerance, that grow in tidal flats, sand dunes, saline depressions, in deserts, or rocky coasts, have the potential to develop several physiological traits [8]. They can attenuate and protect cells from the damage caused by the accumulation of reactive oxygen species (ROS), including superoxide anion (O_2_^−^), singlet oxygen (O_2_), peroxide (O_2_^−2^), hydrogen peroxide (H_2_O_2_), hydroxyl radicals (OH^•^), and hydroxyl (OH^−^) ions and can maintain ion homeostasis [9,10]. In addition, they can promote several biological activities implicated in preventing cancer, chronic inflammation, cardiovascular disorders, and neurodegenerative disease [11,12,13,14]. Oxidative stress has been implicated in Alzheimer’s disease (AD); memory impairment in AD patients is related to the decline in the acetylcholine (ACh) level in the cholinergic system [15]. Therefore, AChE inhibitors are used for stabilizing the ACh neurotransmitter levels in the synaptic cleft [15]. On the other hand, a large number of halophyte plants have been traditionally used to reduce blood pressure (*Salsola kali* L., Chenopodiaceae), for the treatment of cancer (*Artemisia scopariae* Waldst. and Kit., Asteraceae), and microbial infections (e.g., *M. edule*, Aizoaceae) [16] as well as antioxidant, anti-inflammatory, and antitumoral activities [17].

**Figure 1:**
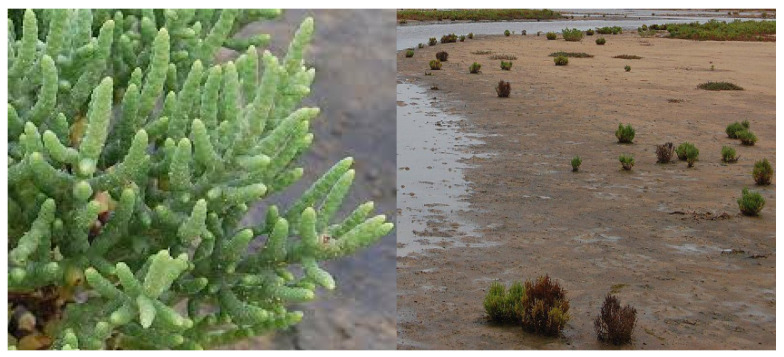
*A. indicum* plant.

*Arthrocnemum indicum* (macrostachyum) (Figure 1) is a stem-succulent perennial, greenish-pinkish, shrubby halophyte plant that belongs to the family of Amaranthaceae (Chenopodiaceae). These species of plant are low shrubs that grow up to 1.5 m, much-branched from the base, and frequently form mats. This plant is abundant in saltmarshes along the coastlines of Europe, South-West Asia, and North Africa [18]. In folkloric medicine, *A. indicum* has been commonly used to treat poisonous bites and stings and possesses beneficial effects against numerous other diseases [16]. The antiproliferative effect of *A. indicum* shoot (leaves and stems) extracts was compared to the control, and the results are very encouraging [8].

In this framework, the aim of the current study was to determine the phytochemical profiling (TPC, TFC, and TCTC) of ethanol, acetone, and hexane *A. indicum* extracts. Then, the phytochemical constituents of the potent(s) extract(s) will be elucidated by HPLC–MS and then correlated to their following pharmacological properties. The antimicrobial, antioxidant, and α-glucosidase activities will be assessed. Finally, the cytotoxicity of the different extracts will also be evaluated using the MTT assay against two cell lines, Hep2 and HT29, and control (Vero).

## 2. Results

### 2.1. Phytochemical Analysis

Regarding the total polyphenols (TPC), flavonoid (TFC), and condensed tannin (TCTC) contents of *A. indicum* shoot extracts, results (Table 1) show that ethanol possesses the highest levels of TPC (303.67 ± 4.16 mg GAE/g DR) and TFC (55.33 ± 2.52 mg CE/g DR) which are significantly different (*p* < 0.05) from acetone and hexane. However, no significant difference (*p* > 0.05) of TCTC between ethanol (11.17 ± 1.26 mg CE/g DR) and acetone (10.33 ± 0.58 mg CE/g DR), but significantly (*p* < 0.05) higher than hexane (2.50 ± 0.50 mg CE/g DR). In contrast, a highly significant difference (*p* < 0.05) of TPC, TFC, and TCTC has been outlined for acetone and hexane.

### 2.2. Polyphenolic Profile

We successfully identified, through the HPLC-MS technique, 19 and 18 phenolic compounds for ethanol and acetone *A. indicum* shoot extracts (Table 2), respectively, with their identities, retention times (Rt), pseudomolecular ions [M-H], and levels. The major compounds for ethanolic extracts followed the order *trans*-ferulic acid (7432.51 ± 27.41 µg/g), *p*-coumaric acid (5982.57 ± 1.37 µg/g), rutin (4108.17 ± 14.31 µg/g cirsiliol (3438.42 ± 19.26 µg/g), 4,5-di-*O*-caffeoylquinic acid (3050.97 ± 8.02 µg/g), hyperoside (quercetin-3-*O*-galactoside) (2067.92 ± 20.65 µg/g), protocatechuic acid (1598.01 ± 1.73 µg/g), and acacetin (882.42 ± 15.58 µg/g); however in acetone, the most predominant compounds were rutin (7987.96 ± 18.73 µg/g) followed, respectively, by 4,5-di-*O*-caffeoylquinic acid 2696.01 ± 24.63 µg/g), hyperoside (quercetin-3-*O*-galactoside) (2513.82 ± 69.82 µg/g), *trans-* ferulic acid (1469.69 ± 36.27 µg/g), *p*-coumaric acid (966.18 ± 32.41 µg/g), acacetin (876.51 ± 26.16 µg/g), and cirsiliol (791.39 ± 2.25 µg/g).

### 2.3. Antioxidant Activity

Extracts rich in phenolics are undoubtedly responsible for hampering oxidative stress via several different mechanisms. To the best of our knowledge, the antioxidant activity of *A. indicum* shoot extracts has not been previously reported. The evaluation of the antioxidant properties of *A. indicum* shoot extracted with three different solvents (hexane, acetone, and ethanol) compared to the authentic standard, BHT, was achieved through 1,1-diphenyl-2-picrylhydrazyl radical scavenging activity (DPPH), Superoxide anion, and ferric reducing antioxidant power (FRAP) methods. As shown (Table 3), the results are displayed as mean ± SD of triplicate tests. Following the DPPH assay, ethanol possesses the strongest ability to reduce the stable radical DPPH to the yellow-colored DPPH-H, displaying an IC_50_ value of 7.17 ± 1.26 μg/mL which is significantly higher than acetone (2.58 times) and hexane (44.76 times) and not significantly (*p* > 0.05) different from the standard, BHT (IC_50_ = 10.70 ± 0.61 μg/mL). Through the superoxide anion test, ethanol exhibited the best antioxidant activity with an IC_50_ of 31.67 ± 1.53 μg/mL (Table 3), which was significantly (*p* < 0.05) more pronounced than that acetone (IC_50_ = 113.67 ± 1.53 μg/mL) and hexane (IC_50_ = 417.00 ± 2.65 μg/mL) but not from the positive control, BHT (IC_50_ = 3.50 ± 0.50 μg/mL). Finally, in the FRAP assay, a potent and significantly different (*p* < 0.05) antioxidant activity was observed with ethanol 80% as compared to those of acetone (1.46 times) and hexane (6.9 times), but still significantly (*p* < 0.05) lower than BHT (23.33 ± 1.53 μg/mL).

Polyphenolics such as TPC and TFC have been considered major contributors to plant antioxidant activities. Quantifications of the main phytochemicals via Pearson’s correlation coefficient (PCC) (Table 4) indicate positive and significantly (*p* < 0.01) and positive correlation to be significant between TPC and TFC (r = 0.994), TPC and TCTC (r = 0.956), and TFC, and TCTC (r = 0.924), justifying that polyphenol contents constitute the most abundant groups. In addition, the antioxidant properties trend was compared to data obtained from the quantifications of the main phytochemicals revealing a negative PCC, meaning that TPC, TFC, and TCTC are the main contributors to the enhancement of the antioxidant activities, which is appointed by lower IC_50_ (DPPH) and/or EC_50_ (FRAP).

### 2.4. Antimicrobial Activity

For bacterial strains, Table 5 shows that IZs are in the range of 8.66 ± 0.57 mm to 14.66 ± 1.50 mm for ethanolic extract, 6.66 ± 0.57 mm to 12.33 ± 0.57 mm for acetone extract, 6.00 ± 0.00 mm to 9.66 ± 0.57 mm for hexane extract, and 16.00 ± 1.00 mm to 27.66 ± 0.57 mm for gentamycin. Concerning fungal strains, IZs are in the range of 12.33 ± 0.57 to 14.00 ± 1.00 for ethanolic extract, 11.00 ± 1.00 to 13.66 ± 0.57 mm for acetone extract, 7.00 ± 1.00 mm to 8.33 ± 0.57 mm for hexane extract, and 16.66 ± 0.57 mm to 19.00 ± 1.00 mm for amphotericin B. According to statistical analysis, *A. indicum* extracts were less efficient than reference antibiotics. In addition, IZs of the three extracts comparison (*p* < 0.05) showed that ethanolic extract was the most active, followed by the acetonic extract, except for the strains *E. feacalis*, *E. coli, L. monocytogenes*, *C. albicans,* and *C. glabrata,* where these two extracts have the same activity. However, hexanoic extract seems less efficient against all tested microorganisms.

For the quantitative method, MIC and MBC values (Table 6) of ethanolic extract (Table 5) were ranged from 0.15 (*M. luteus*) to 1.17 mg/mL (*P. aeruginosa, L. monocytogenes, S. typhimurium*) and from 0.59 (*M. luteus*) to 9.38 mg/mL (*P. aeruginosa*). Concerning acetonic extracts MIC and MBC, values were ranged from 0.59 (*E. feacalis, B. cereus*) to 2.34 mg/mL (*E. coli*, *L. monocytogenes*) and from 2.34 (*M. luteus, E. feacalis*) to 9.38 mg/mL (*E. coli, L. monocytogenes, P. aeruginosa*). Whereas for fungi strains MIC and MFC, values were less. This finding indicates a higher sensitivity level against these two extracts of *A. indicum*. According to these values, the ethanolic extract seems to be more active than the acetonic one.

The ratio MBC/MIC and MFC/MIC (Table 6) has shown a bactericidal effect of the two extracts to all strains tested (except *P. aeruginosa*) and a fungicidal effect for half of the fungal strains. *P. aeruginosa* showed a high level of resistance as with gentamicin.

### 2.5. α-Glucosidase Inhibitory Activity Evaluation

Diabetes, a widespread chronic metabolic disorder in human beings, is characterized by persistent hyperglycemia and disorders of glucose, lipid, and protein metabolism, over-production of free radicals, and oxidative stress. Indeed, in order to reduce the disease burden, natural inhibitors are constantly being sought; one of the main strategies is inhibition of α-glucosidase, which can reduce postprandial hyperglycemia and energy intake, respectively. In this study, the α-glucosidase inhibitory activity of *A. indicum* shoot extracts was evaluated in comparison with a specific standard, acarbose. As can be seen from Table 7, ethanol extract recorded the highest antidiabetic effect with IC_50_ = 13.17 ± 1.04 mg/mL when compared to acetone extract (IC_50_ = 111.50 ± 2.78 mg/mL), and the standard drug, acarbose (IC_50_ = 1.12 ± 0.08 mg/mL) with significant difference (*p* < 0.05).

### 2.6. Cytotoxic Activity

**Figure 2:**
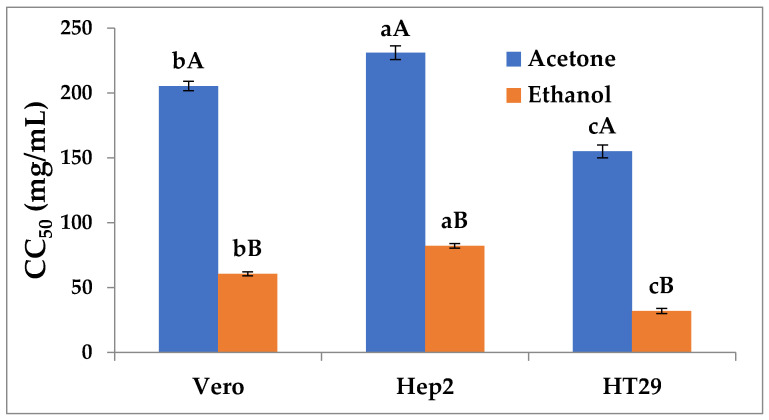
Cytotoxic activity of *A. indicum* extracts against normal and cancer cells. Small letters are used to compare each extract means between different cell lines, while capital letters are used to compare means between extract for the same cell lines.

The anticancer activities of *A. indicum* shoot extracts were evaluated on three human cancer cell lines, including two human tumor cell lines, HT29 and Hep2, and one continuous cell lineage control (Vero) was determined by the MTT. Results (Figure 2) demonstrated an interesting cytotoxic activity against all cancer cell lines with CC_50_ values labeled as the concentration at which 50% of cell growth is inhibited, ranging between 32 mg/mL and 82 mg/mL for ethanol extract and between 155 mg/mL and 231 mg/mL for acetone extract, suggesting that ethanol extract was significantly (*p* < 0.05) more effective than acetone as well as Vero cells. As shown (Figure 2), the HT29 cancer cell line was the most responsive to *A. indicum* shoot extract treatment and significantly (*p* < 0.05) different from the other cancer cells. The promising cytotoxic activity of methanol extract may be explained by its high content of polyphenols.

## 3. Discussion

Plant-based bioactive compounds containing substantial quantities of polyphenols have been gaining much attention nowadays. Our obtained antioxidant results revealed a broad variability in antioxidant values depending on the methods used because antioxidants may exert their effect through various mechanisms. This variability was attributed to the interference of the reaction mechanism and the tested solvents. Typically, the nature of the active molecules present in the samples as well as the presence of phenolic compounds with a certain structure and particular hydroxyl position in the molecule, which can act as a proton donor and show radical scavenging activity. Parallel to that, our antibacterial results showed that Gram-negative bacteria are more resistant than Gram-positive bacteria to the various extracts, especially the ethanol, due to their distinctive structure and to the bacterium’s outer-membrane barrier for Gram-negative bacteria. Their resistance was amplified via chromosomal mutations and lateral gene transfers.

The ethanol extract of *A. indicum* shoots as the most active was dominated by TPC and TFC, which was well supported by LC-MS analysis with the major secondary metabolite being *trans*-ferulic acid with the contents of 7432.51 ± 27.407 µg/g extract, followed by *p*-coumaric acid (5982.57 ± 1.37 µg/g extract), respectively. TPC and TFC are widely present in plant extracts and have been considered significant contributors to their biological activities, exclusively due to their unique redox properties [19]. Therefore, polyphenols containing hydrogen-donating groups have the ability to react with oxidants [20]. Phenolic compounds can also intervene as a potential free radical scavenger by blocking the ROS-induced cytotoxicity and simultaneously decreasing lipid peroxidation and DNA damage [21]. The high level of TPC in ethanol, 80%, might be related to its capacity to solubilize more secondary metabolites displaying a polar character and the higher solubility of a lot of extractable bioactive molecules in this solvent. The highest antioxidant activity of *A. indicum* shoot extracts towards the DPPH test may be due to its polyphenol contents. These two compounds might be greatly involved in the biological activity of this extract, with the others minor by the synergism effect. *Trans*-ferulic acid (4-hydroxy-3-methoxycinnamic acid), which is known for its potent antioxidant activity, is found in many food products and fruits and is used in cosmetology [22]. The safety of ferulic acid has been demonstrated with evidence that a high level of ferulic acid (0.5 and 1 mM) does not affect the cell viability in 786-O human renal cancer cells [23]. Besides that, the anticancer activity of ferulic acid has been proven against different cancer cells, including breast cancer cells (MCF-7) and liver cancer cells (HepG2) [24], human urinary bladder carcinoma (T24) [25], human osteosarcoma (143B and MG63) [26], human breast cancer (MDA-MB-231) [27], and human renal adenocarcinoma (ACHN) cells [28]. Additionally, the inhibition of A549 and HT29-D4 cancer cells was induced by ferulic acid [29]. Ferulic acid has been proven previously for its antioxidant activity, which was mainly related to its resonance stabilization [30]. Ferulic acid helps in neutralizing the free radicals. Bami et al. [30] suggested ferulic acid can hamper oxidative stress and regulate the levels of protein nitrotyrosine, malondialdehyde, blood urea nitrogen, myeloperoxidase, total antioxidant status, and creatinine in rats treated with cisplatin. Alam et al. [31] reported that ferulic acid improves cardiovascular and kidney structure. It was able to decrease the hydrophobicity of *P. aeruginosa* [32]. In the study of Ijabadeniyi et al. [33], ferulic acid was well demonstrated for its antimicrobial activity [33]. Merkl et al. [34] stated that ferulic acid could inhibit the growth of *E. coli*. In addition to all of the above, ferulic acid has been proven for its neuroprotective and antidiabetic properties as well as having high synergistic interaction with hypoglycemic drugs [35,36,37].

The second major identified compound in *A. indicum* shoot ethanol extract, *p*-coumaric acid (4-hydroxycinnamic acid), is a natural ligand abundant in many fruits, vegetables, and cereals with diverse health benefits. The safety of *p*-coumaric acid has been investigated, and the results outlined no significant cytotoxicity [38]. Previous studies have demonstrated the significant relationship between *p*-coumaric acid and antioxidant and antihyperlipidemic activities [39]. The authors suggested that *p*-coumaric is a potent antioxidant with potential therapeutic efficacy for treating hyperlipidemia symptoms [40]. Kilic et al. [39] reported that it is a good scavenger. Besides that, the antimicrobial role of *p*-coumaric acid has been proven. Boz et al. [41] demonstrated the antimicrobial activity of *p*-coumaric acid allows the disrupting of bacterial cell membranes [41]. *p*-coumaric acid was found to inhibit the proliferation and migration of cancer cells [42]. Moreover, the chemopreventive effects of *p*-coumaric acid on colon cancer have been illustrated [43].

The third predominant compound was found to be rutin (3,30,40,5,7-pentahydroxyflavone-3-rhamnoglucoside), which exists in high levels in ethanolic extract (4108.17 ± 14.31 µg/g extract) and acetonic extract (7987.96 ± 18.73 µg/g extract), and must be taken into account. Rutin has been verified for its carcinogenicity, and data showed no carcinogenic potential in non-inbred golden hamsters. In fact, the flavonol rutin has been studied for its antidiabetic effect. It was added for glycemic control by increasing the insulin receptor kinase property [44]. Also, it possesses a protective effect on hepatic and cardiac toxicity [45]. The pharmacological properties of rutin have also been widely studied, including its antileukemic potential [46], anti-inflammatory, antimicrobial, anticarcinogenic, neuroprotective, antithrombotic, and antiviral activities [47,48].

## 4. Materials and Methods

### 4.1. Chemical Reagents

Na_2_CO_3_, Folin–Ciocalteu reagent, gallic acid, NaNO_2_, AlCl_3_, 6H_2_O, vanillin, 2,2-diphenyl- 1picrylhydrazyl (DPPH), NaOH, trichloroacetic acid iron, FeCl_3_, and catechin were purchased from Fluka (Buchs, Switzerland). NBT, NADH, PMS, butylated hydroxytoluene (BHT), Intestinal Alpha-glucosidase type I, acarbose, 4-nitrophenyl β-d-glucopyranoside, MTT (3-[4,5-dimethylthiazol-2-yl]-2,5-diphenyl tetrazolium bromide), and solvents (acetone, ethanol, hexane, methanol, dimethyl sulfoxide (DMSO)) were purchased from Sigma-Aldrich (GmbH, Sternheim, Germany). Mueller-Hinton medium, Sabouraud Chloramphenicol agar, Mueller-Hinton broth, Sabouraud Chloramphenical broth, Gentamycin, and Amphotericin B were purchased from (Bio-Rad^®^, Marnes-la-Coquette, France).

### 4.2. Plant Sampling and Extract Preparation

**Figure 3:**
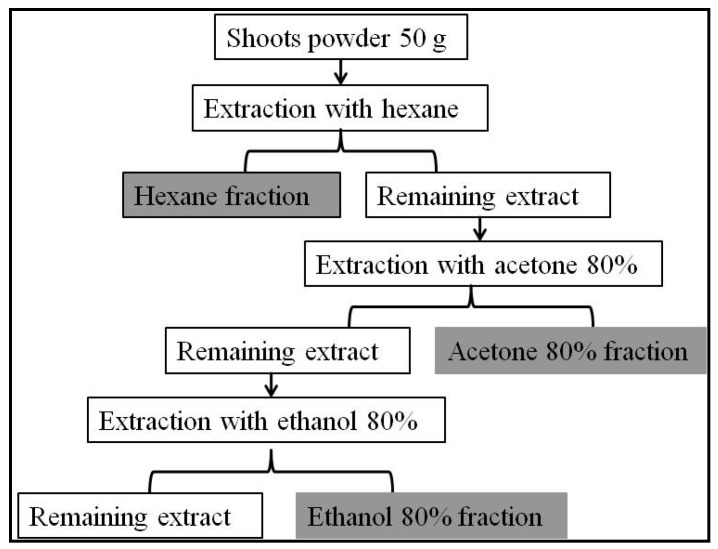
Extraction procedure of *A. indicum* shoots.

The samples of *A. indicum* were collected from Sabkha El-Ogla (35.074594° N; 9.605516° E; semi-arid bioclimatic stage; mean annual rainfall: 200–250 mm/year). The collected *A. indicum* plant was authenticated by Dr. Zouhair Noumi, University of Sfax, Tunisia (Voucher No: H2/200). Plant sampling and extract preparation was referred to in the work of Aouadi et al. [9] (Figure 3).

### 4.3. Colorimetric Quantification of Antioxidants

All samples were analyzed in triplicate.

#### 4.3.1. TPC Assay

Polyphenols were determined, as described by Dewanto et al. [49]. The phenol contents were expressed in terms of milligram gallic acid equivalent per gram of dry residue (mg GAE/g DR).

#### 4.3.2. TFC Assay

Total flavonoids were measured colorimetrically according to Dewanto et al. [49]. Total flavonoid content was expressed as mg catechin per gram of DR (mg CE/g DR).

#### 4.3.3. TCTC Assay

The analysis of condensed tannins was carried out according to the method of Sun et al. [50]. The amount of total condensed tannins was expressed as mg (+)-catechin equivalent/g DR.

### 4.4. HPLC-MS Analysis of Phenolic Compounds

The identification of polyphenolics was done using the Shimadzu HPLC-MS 2020 system. Detailed experiments were the same as per the reported method of Hajlaoui et al. [11]. Phenolic acids and flavonoids present in the extracts were identified by comparison of their *m/z* of [M-H]- fragment in mode SIM and retention times with those of 33 standards available in the laboratory. Standards were purchased.

### 4.5. Antioxidant Activity

The DPPH quenching ability of the extract was measured according to the same experiment as described by Felhi et al. [6]. Superoxide anion scavenging activity was assessed using the method described by Saini et al. [51]. The ability of these extracts to reduce Fe^3+^ via FRAP test was assayed using the method described by Bakari et al. [14].

### 4.6. Antimicrobial Activity

#### 4.6.1. Microorganisms

The bacterial species consisted of 4 Gram-positive and 4 Gram-negative bacterial strains. The fungal species belonged to 4 ATCC Candida strains.

#### 4.6.2. Disc-Diffusion Assay

Antimicrobial activity testing was performed according to the protocol described by and slightly modified by Hajlaoui et al. [52,53] and Snoussi et al. [54], and Ingkaninan et al. [55]. Gentamycin (10 μg/disc) and Amphotericin B (20 μg/disc) were used as a positive reference.

#### 4.6.3. Micro-Well Determination of MIC, MBC, and MFC

Minimal inhibition concentration (MIC), minimal bactericidal concentration (MBC), and minimal fungicidal concentration (MFC) values were also determined. For bacterial strains, we used MHB (Mueller-Hinton broth), and for yeast, we used SCB (Sabouraud Chloramphenicol broth).

### 4.7. Cytotoxicity Assay

The cytotoxic effects of the samples were evaluated based on the reduction of MTT (3-[4,5-dimethylthiazol-2-yl]- 2,5-diphenyl tetrazolium bromide) by the mitochondrial dehydrogenase of viable cells to give a blue formazan product that can be measured spectrophotometrically at 540 nm [56]. Data were obtained from duplicate wells.

### 4.8. α-Glucosidase Inhibitory Assay

The α-glucosidase assay of the tested extracts was conducted according to the standard method with slight modification [57].

### 4.9. Statistical Analysis

Differences in means were calculated using Duncan’s multiple range tests for means with a 95% confidence interval (*p* ≤ 0.05).

## 5. Conclusions

The current study showed for the first time that A. indicum shoot extracts possess considerable biological activities, with ethanolic extract being found to be the most active. The latter was characterized by its high content of trans-ferulic acid, *p*-coumaric acid, and rutin. Moreover, significant differences in in vitro antioxidant, antimicrobial, antidiabetic, and cytotoxicity activities have been recorded. As compared to the positive control, ethanolic extract displayed excellent scavenging ability towards the DPPH test, moderate scavenging against the superoxide anion test, and good antioxidant power with the FRAP assay, with the strongest correlation found between phytochemicals and antioxidant potency. Bactericidal effect of the two extracts to all tested strains (except P. aeruginosa) and a fungicidal effect for most of the fungal strains have been shown. The examination of the antidiabetic effect confirmed the potent α-glucosidase inhibitory effect of ethanol extract, which is 8.4 times higher than acetone extract. Cytotoxicity results also outlined the high efficacy of ethanol extract to be more responsive to *A. indicum* shoot against the two tested cell lines, HT29 in a level of 4.8 times and Hep2 in a level of 2.8 times, when compared to the acetone extract, respectively. Also, HT29 exhibited a higher cytotoxicity level than the control Vero cell lines, which is about half-fold. As a future thrust, studies should be conducted on ethanolic extract of A. indicum shoot extract as a starting point to carry out further in vivo studies. These findings suggest that *A. indicum* ethanol extract could be a promising antioxidant, antimicrobial, antidiabetic, and cytotoxic agent.

## Figures and Tables

**Table 1:** TPC, TFC and TCTC of *A. indicum* shoot extracts.

Fractions	TPC (mg GAE/g DR)	TFC (mg CE/g DR)	TCTC (mg CE/g DR)
Ethanol	303.67 ± 4.16 ^a^	55.33 ± 2.52 ^a^	11.17 ± 1.26 ^a^
Acetone	207.00 ± 4.00 ^b^	36.17 ± 1.04 ^b^	10.33 ± 0.58 ^a^
Hexane	16.00 ± 1.73 ^c^	6.17 ± 1.26 ^c^	2.50 ± 0.50 ^b^

Means (three replicates) followed by at least one same letter within a row are not significantly different at *p* < 0.05.

**Table 2:** Identified by acetone and ethanol extract obtained from *A. indicum* shoots.

Peaks	Compounds	MS [M−H]^−^*m/z*	Retention Time (min)	Quantity in µg/g Extract	
				Ethanol	Acetone
1	Quinic acid	191.00	2130	305.62 ± 9.62	287.90 ± 12.81
2	Protocatchuic acid	153.00	7385	1598.01 ± 1.73	343.01 ± 6.53
3	Epicatechin	289.00	13.795	54.48 ± 2.22	-
4	4-*O*-caffeoylquinic acid	353.00	12.562	646.71 ± 5.50	437.72 ± 0.20
5	Caffeic acid	179.00	12.993	82.99 ± 2.04	115.58 ± 4.88
6	1,3-di-*O*-caffeoyquinic acid	515.00	14.960	198.45 ± 8.14	118.46 ± 1.20
7	*p*-Coumaric acid	163.00	17.087	5982.57 ± 1.37	966.18 ± 32.41
8	*Trans*-Ferulic acid	193.00	18.744	7432.51 ± 27.41	1469.69 ± 36.27
9	Rosmarinic acid	359.00	22.209	259.42 ± 2.98	58.13 ± 3.27
10	Hyperoside (quercetin-3-*O*-galactoside)	463.00	22.910	2067.92 ± 20.65	2513.82 ± 69.82
11	Rutin	609.00	23.136	4108.17 ± 14.31	7987.96 ± 18.73
12	Salvianolic acid	717.00	23.762	174.17 ± 1.91	70.40 ± 1.19
13	4,5-di-*O*-caffeoylquinic acid	515.00	23.902	3050.97 ± 8.02	2696.01 ± 24.63
14	Quercetrin (quercetin-3-*O*-Rhamonoside)	447.00	25.112	212.34 ± 1.677	133.02 ± 1.49
15	Naringenin	271.00	26.977	492.82 ± 7.40	94.32 ± 1.00
16	Silymarin	481.00	28.852	129.87 ± 2.31	50.72 ± 1.44
17	Apegenin	269.00	32.391	49.84 ± 1.15	24.40 ± 2.03
18	Cirsiliol	329.00	32.451	3438.42 ± 19.26	791.39 ± 2.25
19	Acacetin	283.00	37.061	882.42 ± 15.58	876.51 ± 26.16

**Table 3:** DPPH radical-scavenging activity, superoxide anion radical-scavenging activity, and FRAP assays. Means (three replicates) followed by at least one same letter within a row are not significantly different at *p* < 0.05.

Fractions	DPPH (IC_50_ μg/mL)	Superoxide Anion (IC_50_ μg/mL)	Reducing Power (EC_50_ μg/mL)
Ethanol	7.17 ± 1.26 ^c^	31.67 ± 1.53 ^c^	51.67 ± 1.53 ^c^
Acetone	18.50 ± 1.80 ^b^	113.67 ± 1.53 ^b^	75.67 ± 2.08 ^b^
Hexane	321.00 ± 3.61 ^a^	417.00 ± 2.65 ^a^	356.67 ± 2.08 ^a^
BHT	10.70 ± 0.61 ^c^	3.50 ± 0.50 ^d^	23.33 ± 1.53 ^d^

**Table 4:** Pearson’s Correlation.

	TPC	TFC	CTC	DPPH	Superoxide Anion	FRAP
TPC	1					
TFC	0.994 **	1				
TCTC	0.956 **	0.924 **	1			
DPPH	−0.953 **	−0.932 **	−0.983 **	1		
Superoxide Anion	−0.991 **	−0.979 **	−0.978 **	0.985 **	1	
FRAP	−0.965 **	−0.945 **	−0.984 **	0.999 **	0.991 **	1

**. Correlation is significant at 0.01 level (bilateral).

**Table 5:** Inhibition zones of growth (IZ mm ± SD), showing the qualitative antimicrobial activity of three *A. indicum* extracts against human pathogenic bacteria compared to standard antibiotics (Gentamycin, Amphotericin B).

Strains	Ethanol	Acetone	Hexane	Antibiotics
Gram-positive bacteria				Gent.
*S. epidermidis* CIP 106510	14.66 ± 1.15 ^aB^	11.66 ± 0.57 ^aC^	7.00 ± 1.00 ^bcD^	22.00 ± 1.00 ^bcA^
*M. luteus* NCIMB 8166	14.00 ± 0.00 ^aB^	12.00 ± 1.00 ^aC^	8.00 ± 00 ^bD^	27.50 ± 0.50 ^aA^
*E. feacalis* ATCC 29212	14.16 ± 1.25 ^aB^	12.33 ± 0.57 ^aB^	8.00 ± 1.00 ^bC^	26.00 ± 1.00 ^aA^
*B. cereus* ATCC 14579	12.33 ± 0.57 ^bB^	11.00 ± 1.00 ^abC^	9.66 ± 0.57 ^aD^	27.66 ± 0.57 ^aA^
**Gram-negative bacteria**				
*E. coli* ATCC 35218	11.33 ± 0.57 ^bcB^	11.33 ± 1.15 ^aB^	7.00 ± 0.00 ^bcC^	21.66 ± 0.57 ^bcA^
*L. monocytogenes* ATCC19115	10.66 ± 0.57 ^cB^	9.66 ± 0.57 ^bB^	7.66 ± 0.57 ^bC^	23.00 ± 1.00 ^bA^
*P. aeruginosa* ATCC 27853	8.66 ± 0.57 ^cdB^	6.66 ± 0.57 ^cC^	6.00 ± 0.00 ^cC^	16.00 ± 1.00 ^dA^
*S. typhimurium* LT2 DT104	10.00 ± 1.00 ^dB^	7.66 ± 0.57 ^cC^	7.66 ± 0.57 ^bC^	20.66 ± 1.52 ^cA^
**Fungal strains**				**Amph.**
*Candida albicans ATCC 90028*	12.33 ± 0.57 ^bB^	12.33 ± 0.57 ^abB^	8.00 ± 0.00 ^abC^	19.00 ± 1.00 ^aA^
*Candida glabrata ATCC 90030*	13.66 ± 0.57 ^abB^	13.33 ± 0.57 ^aB^	7.00 ± 1.00 ^bC^	16.66 ± 0.57 ^bA^
*Candida parapsilosis ATCC 22019*	14.00 ± 1.00 ^aB^	11.66 ± 1.52 ^abC^	7.66 ± 0.57 ^abD^	18.33 ± 0.57 ^aA^
*Candida krusei ATCC 6258*	12.66 ± 0.57 ^abB^	11.00 ± 1.00 ^bC^	8.33 ± 0.57 ^aD^	18.00 ± 1.00 ^abA^

SD: Standard deviation; IZ: Inhibition zone diameter (mm) around the discs (6 mm) impregnated with 10 μL of extract and 10 μg/disc for Gentamycin (Gent); a, b, c, d, A, B, C, D: Each value represents the average of 3 repetitions. Means followed by the same letters are not significantly different at *p* < 0.05 based on Duncan’s multiple range test. Small letters are used to compare each extract means between different strains, while capital letters are used to compare means between extract for the same strain.

**Table 6:** Minimal inhibition concentration (MIC mg/mL), minimal bactericidal concentration (MBC mg/mL), minimal fungicidal concentration (MFC mg/mL), and ratios (MBC/MIC and MFC/MIC) showing quantitative antimicrobial activity of two *A. indicum* extracts (ethanol and acetone) against human pathogenic bacteria and fungus compared to standard antibiotics (Gentamycin and Amphotericin B).

	Ethanol			Acetone			Antibiotics		
	MIC	MBC	MBC/MIC	MIC	MBC	MBC/MIC	MIC	MBC	MBC/MIC
Bacterial strains							Gentamycin		
*S. epidermidis* CIP 106510	0.29	1.17	4 (Bactericidal)	1.17	4.69	4 (Bactericidal)	0.009	0.039	4 (Bactericidal)
*M. luteus* NCIMB 8166	0.15	0.59	4 (Bactericidal)	1.17	4.69	4 (Bactericidal)	0.004	0.019	4 (Bactericidal)
*E. feacalis* ATCC 29212	0.29	1.17	4(Bactericidal)	0.59	2.34	2 (Bactericidal)	0.004	0.019	4 (Bactericidal)
*B. cereus* ATCC 14579	0.29	1.17	4 (Bactericidal)	0.59	2.34	2 (Bactericidal)	0.004	0.039	4 (Bactericidal)
*E. coli* ATCC 35218	1.17	4.69	4 (Bactericidal)	2.34	9.38	4 (Bactericidal)	0.004	0.039	4 (Bactericidal)
*L. monocytogenes* ATCC19115	1.17	4.69	4 (Bactericidal)	2.34	9.38	2 (Bactericidal)	0.019	0.078	4 (Bactericidal)
*P. aeruginosa* ATCC 27853	1.17	9.38	8(Bacteriostatic)	1.17	9.38	8 (Bacteriostatic)	0.019	0.15	8 (Bacteriostatic)
*S. typhimurium* LT2 DT104	0.59	2.34	4 (Bactericidal)	1.17	4.69	2 (Bactericidal)	0.019	0.039	2 (Bactericidal)
**Fungal strains**		**MFC**	**MFC/MIC**		**MFC**	**MFC/MIC**	**Amphotericin B** **MFC MFC/MIC**		
*C. albicans ATCC 90028*	0.15	1.17	8 (fungistatic)	0.59	4.69	8 (fungistatic)	0.078	0.31	4 (Fungicidal)
*C. glabrata ATCC 90030*	0.15	0.59	4 (Fungicidal)	0.59	2.34	4 (Fungicidal)	0.078	0.31	4 (Fungicidal)
*C. parapsilosis ATCC 22019*	0.15	0.59	4 (Fungicidal)	0.59	2.34	4 (Fungicidal)	0.039	0.078	2 (Fungicidal)
*C. krusei ATCC 6258*	0.15	1.17	8 (fungistatic)	0.59	4.69	8 (fungistatic)	0.078	0.31	4 (Fungicidal)

**Table 7:** Inhibitory activity of α-glucosidase of two *A. indicum* extracts compared to authentic standard (Acarbose).

	α-Glucosidase (IC_50_ mg/mL)
Ethanol	13.17 ± 1.04 ^b^
Acetone	111.50 ± 2.78 ^a^
Acarbose	1.12 ± 0.08 ^c^

Means (three replicates) followed by at least one same letter are not significantly different at *p* < 0.05.

## Data Availability

All data generated or analyzed during this study are included in this article.

## References

[1] Ben Mefteh F., Daoud A., Bouket A.C., Thissera B., Kadri Y., Cherif-Silini H., Eshelli M., Alenezi F.N., Vallat A., Oszako T. (2018). Date Palm Trees Root-Derived Endophytes as Fungal Cell Factories for Diverse Bioactive Metabolites. Int. J. Mol. Sci..

[2] Hajlaoui H., Arraouadi S., Noumi E., Aouadi K., Adnan M., Khan M.A., Kadri A., Snoussi M. (2021). Antimicrobial, Anti-oxidant, Anti-Acetylcholinesterase, Antidiabetic, and Pharmacokinetic Properties of *Carum carvi* L. and *Coriandrum sativum* L. Essential Oils Alone and in Combination. Molecules.

[3] Mseddi K., Alimi F., Noumi E., Veettil V.N., Deshpande S., Adnan M., Hamdi A., Elkahoui S., Alghamdi A., Kadri A. (2020). *Thymus musilii* Velen. as a promising source of potent bioactive compounds with its pharmacological properties: In vitro and in silico analysis. Arab. J. Chem..

[4] Daoud A., Ben Mefteh F., Mnafgui K., Turki M., Jmal S., Ben Amar R., Ayadi F., ElFeki A., Abid L., Rateb M.E. (2017). Cardiopreventive effect of ethanolic extract of date palm pollen against isoproterenol induced myocardial infarction in rats through the inhibition of the angiotensin-converting enzyme. Exp. Toxicol. Pathol..

[5] Gad-Elkareem M.A.M., Abdelgadir E.H., Badawy O.M., Kadri A. (2019). Potential antidiabetic effect of ethanolic and aqueous-ethanolic extracts of *Ricinus communis* leaves on streptozotocin-induced diabetes in rats. PeerJ.

[6] Felhi S., Hajlaoui H., Ncir M., Bakari S., Ktari N., Saoudi M., Gharsallah N., Kadri A. (2016). Nutritional, phytochemical and antioxidant evaluation and FT-IR analysis of freeze-dried extracts of *Ecballium elaterium* fruit juice from three localities. Food Sci. Technol..

[7] Felhi S., Daoud A., Hajlaoui H., Mnafgui K., Gharsallah N., Kadri A. (2017). Solvent extraction effects on phytochemical constituents profiles, antioxidant and antimicrobial activities and functional group analysis of *Ecballium elaterium* seeds and peels fruits. Food Sci. Technol..

[8] Ksouri R., Ksouri W.M., Jallali I., Debez A., Magné C., Hiroko I., Abdelly C. (2012). Medicinal halophytes: Potent source of health promoting biomolecules with medical, nutraceutical and food applications. Crit. Rev. Biotechnol..

[9] Aouadi K., Hajlaoui H., Arraouadi S., Ghannay S., Snoussi M., Kadri A. (2021). HPLC/MS Phytochemical Profiling with Antioxidant Activities of *Echium humile* Desf. Extracts: ADMET Prediction and Computational Study Targeting Human Peroxiredoxin 5 Receptor. Agronomy.

[10] Sajkowska-Kozielewicz J.J., Kozielewicz P., Barnes N.M., Wawer I., Paradowska K. (2016). Antioxidant, cytotoxic, and antiproliferative activities and total polyphenol contents of the extracts of *Geissospermum reticulatum* bark. Oxid. Med. Cell. Longev..

[11] Hajlaoui H., Arraouadi S., Mighri H., Chaaibia M., Gharsallah N., Ros G., Nieto G., Kadri A. (2019). Phytochemical Constituents and Antioxidant Activity of Oudneya Africana L. Leaves Extracts: Evaluation Effects on Fatty Acids and Proteins Oxidation of Beef Burger during Refrigerated Storage. Antioxidants.

[12] Felhi S., Saoudi M., Daoud A., Hajlaoui H., Ncir M., Chaabane R., El Feki A., Gharsallah N., Kadri A. (2017). Investigation of phytochemical contents, in vitro antioxidant and antibacterial behavior and in vivo anti-inflammatory potential of *Ecballium elaterium* methanol fruits extract. Food Sci. Technol..

[13] Bakari S., Hajlaoui H., Daoud A., Mighri H., Ross-Garcia J.M., Gharsallah N., Kadri A. (2018). Phytochemicals, antioxidant and antimicrobial potentials and LC-MS analysis of hydroalcoholic extracts of leaves and flowers of *Erodium glaucophyllum* collected from Tunisian Sahara. Food Sci. Biotechnol..

[14] Bakari S., Daoud A., Felhi S., Smaoui S., Gharsallah N., Kadri A. (2017). Proximate analysis, mineral composition, phytochemical contents, antioxidant and antimicrobial activities and GC-MS investigation of various solvent extracts of cactus cladode. Food Sci. Technol..

[15] Takomthong P., Waiwut P., Yenjai C., Sombatsri A., Reubroycharoen P., Lei L., Lai R., Chaiwiwatrakul S., Boonyarat C. (2021). Multi-Target Actions of Acridones from *Atalantia monophylla* towards Alzheimer’s Pathogenesis and Their Pharmacokinetic Properties. Pharmaceuticals.

[16] Hurtado-Fernandez E., Pacchiarotta T., Mayboroda O.A., Fernandez-Gutierrez A., Carrasco-Pancorbo A. (2014). Quantitative characterization of important metabolites of avocado fruit by gas chromatography coupled to different detectors (APCI-TOF MS and FID). Food Res. Int..

[17] Liebezeit G., Künnemann T.D., Gad G. (1999). Biotechnological potential of North sea salt marsh plants-a review of traditional knowledge. Prog. Ind. Microbiol..

[18] Redondo-Gómez S., Mateos-Naranjo E., Figueroa M.E., Davy A.J. (2010). Salt stimulation of growth and photosynthesis in an extreme halophyte Arthrocnemum macrostachyum. Plant Biol..

[19] Muflihah Y.M., Gollavelli G., Ling Y.-C. (2021). Correlation Study of Antioxidant Activity with Phenolic and Flavonoid Compounds in 12 Indonesian Indigenous Herbs. Antioxidants.

[20] Kumar A., Kaushik P., Incerpi S., Pedersen J.Z., Goel S., Prasad A.K., Rohil V., Parmar V.S., Saso L., Len C. (2021). Evaluation of the Free Radical Scavenging Activities of Ellagic Acid and Ellagic Acid Peracetate by EPR Spectrometry. Molecules.

[21] Majtan J. (2014). Honey: An immunomodulator in wound healing. Wound Repair Regen..

[22] Kelainy E.G., Laila I.M.I., Ibrahim S.R. (2019). The effect of ferulic acid against lead-induced oxidative stress and DNA damage in kidney and testes of rats. Environ. Sci. Poll. Res..

[23] Caparica R., Júlio A., Baby A.R., de Almeida T.S., Costa J.G. (2020). In vitro cytotoxicity assessment of ferulic, caffeic and p-coumaric acids on human renal cancer cells. Biomed. Biopharm. Res..

[24] ElKhazendar M., Chalak J., El-Huneidi W., Vinod A., Abdel-Rahman W.M., Abu-Gharbieh E. (2019). Antiproliferative and proapoptotic activities of ferulic acid in breast and liver cancer cell lines. Trop. J. Pharm. Res..

[25] Peng C.C., Chyau C.C., Wang H.E., Chang C.H., Chen K.C., Chou K.Y., Peng R.Y. (2013). Cytotoxicity of ferulic acid on T24 cell line differentiated by different microenvironments. BioMed Res. Int..

[26] Wang T., Gong X., Jiang R., Li H., Du W., Kuang G. (2016). Ferulic acid inhibits proliferation and promotes apoptosis via blockage of PI3K/Akt pathway in osteosarcoma cell. Am. J. Transl. Res..

[27] Zhang X., Lin D., Jiang R., Li H., Wan J., Li H. (2016). Ferulic acid antitumor activity and inhibits metastasis in breast cancer cells by regulating epithelial to mesenchymal transition. Oncol. Rep..

[28] Karimvand M.N., Kalantar H., Khodayar M.J. (2021). Cytotoxic and apoptotic effects of ferulic acid on renal carcinoma cell line (ACHN). Jundishapur J. Nat. Pharm. Prod..

[29] Bouzaiene N.N., Kilani Jaziri S., Kovacic H., Chekir-Ghedira L., Ghedira K., Luis J. (2015). The effects of caffeic, coumaric and ferulic acids on proliferation, superoxide production, adhesion and migration of human tumor cells in vitro. Eur. J. Pharmacol..

[30] Bami E., Ozakpinar O.B., Ozdemir-Kumral Z.N., Köroglu K., Ercan F., Cirakli Z., Sekerler T., Izzettin F.V., Sancar M., Okuyan B. (2017). Protective effect of ferulic acid on cisplatin induced nephrotoxicity in rats. Environ. Toxicol. Pharmacol..

[31] Alam M.A., Sernia C., Brown L. (2013). Ferulic Acid Improves Cardiovascular and Kidney Structure and Function in Hypertensive Rats. J. Cardiovasc. Pharmacol..

[32] Borges A., Ferreira C., Saavedra M.J., Simões M. (2013). Antibacterial Activity and Mode of Action of Ferulic and Gallic Acids Against Pathogenic Bacteria. Microb. Drug Resist..

[33] Ijabadeniyi O.A., Govender A., Olagunju O.F., Oyedeji A.B. (2021). The antimicrobial activity of two phenolic acids against foodborne Escherichia coli and Listeria monocytogenes and their effectiveness in a meat system. Ital. J. Food Sci..

[34] Merkl R., Hrádková I., Filip V., Šmidrkal J. (2010). Antimicrobial and antioxidant properties of phenolic acids alkyl esters. Czech J. Food Sci..

[35] Ren Z., Zhang R., Li Y., Li Y., Yang Z., Yang H. (2017). Ferulic acid exerts neuroprotective effects against cerebral ischemia/reperfusion–induced injury via antioxidant and anti-apoptotic mechanisms in vitro and in vivo. Int. J. Mol. Med..

[36] Narasimhan A., Chinnaiyan M., Karundevi B. (2015). Ferulic acid exerts its antidiabetic effect by modulating insulin-signalling molecules in the liver of high-fat diet and fructose-induced type-2 diabetic adult male rat. Appl. Physiol. Nutr. Metab..

[37] Prabhakar P.K., Prasad R., Ali S., Doble M. (2013). Synergistic interaction of ferulic acid with commercial hypoglycemic drugs in streptozotocin induced diabetic rats. Phytomedicine.

[38] Boo Y.C. (2019). *p*-Coumaric Acid as An Active Ingredient in Cosmetics: A Review Focusing on its Antimelanogenic Effects. Antioxidants.

[39] Yingbin S., Xun S., Li L., Jian S., Yogini J., Junqing H., Chun L., Wenjian Y., Leonard W., Hui Z. (2019). Protective effects of p-coumaric acid against oxidant and hyperlipidemia-an in vitro and in vivo evaluation. Biomed. Pharmacother..

[40] Kilic I., Yesiloglu Y. (2013). Spectroscopic studies on the antioxidant activity of p-coumaric acid. Spectrochim. Acta Part A Mol. Biomol. Spectrosc..

[41] Boz H. (2015). *p*-Coumaric acid in cereals: Presence, antioxidant and antimicrobial effects. Int. J. Food Sci. Technol..

[42] Roy N., Narayanankutty A., Nazeem P.A., Valsalan R., Babu T.D., Mathew D. (2016). Plant Phenolics Ferulic Acid and P-Coumaric Acid Inhibit Colorectal Cancer Cell Proliferation through EGFR Down-Regulation. Asian Pac. J. Cancer Prev..

[43] Sharma S.H., Rajamanickam V., Nagarajan S. (2018). Antiproliferative effect of *p*-Coumaric acid targets UPR activation by downregulating Grp78 in colon cancer. Chem. Biol. Interact..

[44] Hsu C.Y., Shih H.Y., Chia Y.C., Lee C.H., Ashida H., Lai Y.K., Weng C.F. (2014). Rutin potentiates insulin receptor kinase to enhance insulin-dependent glucose transporter 4 translocation. Mol. Nutr. Food Res..

[45] Fernandes A.A., Novelli E.L., Okoshi K., Okoshi M.P., Di Muzio B.P., Guimaraes J.F., Fernandes Junior A. (2010). Influence of rutin treatment on biochemical alterations in experimental diabetes. Biomed. Pharmacother..

[46] Lin J.P., Yang J.S., Lin J.J., Lai K.C., Lu H.F., Ma C.Y., Sai-Chuen Wu R., Wu K.C., Chueh F.S., Gibson Wood W. (2012). Rutin inhibits human leukemia tumor growth in a murine xenograft model in vivo. Environ. Toxicol..

[47] Agrawal P.K., Agrawal C., Blunden G. (2021). Rutin: A potential antiviral for repurposing as a SARS-CoV-2 main protease (M^pro^) inhibitor. Nat. Prod. Commun..

[48] Ganeshpurkar A., Saluja A.K. (2017). The pharmacological potential of rutin. Saudi Pharm. J..

[49] Dewanto V., Wu X., Adom K.K., Liu R.H. (2002). Thermal processing enhances the nutritional value of tomatoes by increasing total antioxidant activity. J. Agric. Food Chem..

[50] Sun B., Richardo-da-Silvia M., Spranger I. (1998). Critical factors of vanillin assay for catechins and proanthocyanidins. J. Agric. Food Chem..

[51] Saini A., Pandey A., Sharma S., Suradkar U.S., Ambedkar Y.R., Meena P., Raman R., Gurjar A.S. (2020). Assessment of antioxidant activity of rosemary (*Rosmarinus officinalis*) leaves extract. J. Pharmacogn. Phytochem..

[52] Kadri A., Zarai Z., Ben Chobba I., Gharsallah N., Damak M., Bekir A. (2011). Chemical composition and in vitro antioxidant activities of *Thymelaea hirsuta* L. essential oil from Tunisia. Afri. J. Biotechnol..

[53] Hajlaoui H., Snoussi M., Noumi E., Zanetti S., Ksouri R., Bakhrouf A. (2010). Chemical composition. antioxidant and antibacterial activities of the essential oils of five Tunisian aromatic plants. Ital. J. Food Sci..

[54] Snoussi M., Hajlaoui H., Noumi E., Usai D., Sechi L.A., Zanetti S., Bakhrouf A. (2008). In-vitro anti-*Vibrio* spp. activity and chemical composition of some Tunisian aromatic plants. World J. Microbiol. Biotechnol..

[55] Ingkaninan K., Temkittawon P., Chuenchon K., Yuyaem T., Thongnoi W. (2003). Screening for acetylcholinesterase inhibito activity in plants used in Thai traditional rejuvenating and neurotonic remedies. J. Ethnopharmacol..

[56] Magadula J.J., Sulmimani H.O. (2010). Cytotoxic and anti-HIV activities of some Tanzanian Garcinia species. Tanzan. J. Health Res..

[57] Asghari B., Salehi P., Sonboli A., Ebrahimi S.N. (2015). Flavonoids from Salvia chloroleuca with alpha-amylsae and alpha-glucosidase inhibitory effect. Iran. J. Pharm. Res..

